# The Effect of Oxidized Dopamine on the Structure and Molecular Chaperone Function of the Small Heat-Shock Proteins, αB-Crystallin and Hsp27

**DOI:** 10.3390/ijms22073700

**Published:** 2021-04-02

**Authors:** Junna Hayashi, Jennifer Ton, Sparsh Negi, Daniel E. K. M. Stephens, Dean L. Pountney, Thomas Preiss, John A. Carver

**Affiliations:** 1 Research School of Chemistry, The Australian National University, Acton, ACT 2601, Australia; junna.hayashi@anu.edu.au (J.H.); jennifer.ton@anu.edu.au (J.T.); sparshnegi7@gmail.com (S.N.); DEKMS@hotmail.co.nz (D.E.K.M.S.); 2 Department of Biochemical Engineering and Biotechnology, Indian Institute of Technology Delhi, Hauz Khas, New Delhi 110016, India; 3 School of Medical Science, Griffith University, Gold Coast, QLD 4215, Australia; d.pountney@griffith.edu.au; 4 Department of Genome Sciences, John Curtin School of Medical Research, The Australian National University, Acton, ACT 2601, Australia; thomas.preiss@anu.edu.au; 5 Victor Chang Cardiac Research Institute, Sydney, NSW 2010, Australia

**Keywords:** small heat-shock protein, αB-crystallin, Hsp27, molecular chaperone, Parkinson’s disease, dopamine, oxidation, cross-linking, post-translational modification

## Abstract

Oxidation of the neurotransmitter, dopamine (DA), is a pathological hallmark of Parkinson’s disease (PD). Oxidized DA forms adducts with proteins which can alter their functionality. αB-crystallin and Hsp27 are intracellular, small heat-shock molecular chaperone proteins (sHsps) which form the first line of defense to prevent protein aggregation under conditions of cellular stress. In vitro, the effects of oxidized DA on the structure and function of αB-crystallin and Hsp27 were investigated. Oxidized DA promoted the cross-linking of αB-crystallin and Hsp27 to form well-defined dimer, trimer, tetramer, etc., species, as monitored by SDS-PAGE. Lysine residues were involved in the cross-links. The secondary structure of the sHsps was not altered significantly upon cross-linking with oxidized DA but their oligomeric size was increased. When modified with a molar equivalent of DA, sHsp chaperone functionality was largely retained in preventing both amorphous and amyloid fibrillar aggregation, including fibril formation of mutant (A53T) α-synuclein, a protein whose aggregation is associated with autosomal PD. In the main, higher levels of sHsp modification with DA led to a reduction in chaperone effectiveness. In vivo, DA is sequestered into acidic vesicles to prevent its oxidation and, intracellularly, oxidation is minimized by mM levels of the antioxidant, glutathione. In vitro, acidic pH and glutathione prevented the formation of oxidized DA-induced cross-linking of the sHsps. Oxidized DA-modified αB-crystallin and Hsp27 were not cytotoxic. In a cellular context, retention of significant chaperone functionality by mildly oxidized DA-modified sHsps would contribute to proteostasis by preventing protein aggregation (particularly of α-synuclein) that is associated with PD.

## 1. Introduction

Protein homeostasis, or proteostasis, refers to the cell’s inherent biological pathways and networks which ensure that proteins acquire their native and functional form with their levels being maintained within a strict regime to ensure optimal cell functionality [1,2]. When proteostasis is compromised, proteins can partially unfold, misfold, and aggregate, leading to a plethora of deleterious consequences including cell death [3]. Proteostasis dysregulation underlies more than 55 human disorders including type-II diabetes, cataract, Alzheimer’s disease and Parkinson’s disease (PD) [4]. Proteostasis is synergistically regulated by the protein synthesis machinery, protein degradation pathways and molecular chaperone proteins [1].

Small heat-shock proteins (sHsps) are a family of intracellular molecular chaperone proteins [5]. They interact with and stabilize misfolded and partially unfolded proteins to prevent their aggregation [5]. sHsps are constitutively expressed and are upregulated under stress conditions such as elevated temperature. They do not require ATP to function, enabling them to operate in low-energy cellular stress environments [5]. Most sHsps also have broad target or client protein specificity to prevent protein aggregation, predominantly via hydrophobic interactions [5,6,7]. sHsps are regarded as the cell’s first line of defense against protein aggregation [5,6].

There are ten human sHsps, of which αB-crystallin (αBc or HspB5) and Hsp27 (or HspB1) are the most abundant and ubiquitously expressed [5,6,7]. In solution, αBc and Hsp27 exist as large, heterogeneous oligomers, with an average mass of 650 [8] and 500 kDa [9], respectively. Their monomeric subunit mass is 20.2 and 22.8 kDa [10], respectively. As with all sHsps, the amino acid sequence of αBc and Hsp27 is organized into three regions: the N-terminal region (NTR), the α-crystallin domain (ACD) and the C-terminal region (CTR). The NTR is variable in length, mostly unstructured and is required to form large sHsp oligomers [11]. In contrast, the central ACD is highly conserved and well structured. It is comprised of antiparallel β-sheet strands arranged in an immunoglobulin fold, and forms a dimer in the absence of the terminal regions [12]. On its own, the ACD possesses chaperone ability [13]. The dimer is the basic building block of the intact sHsp oligomer [10,13,14]. The unstructured CTR provides solubility to the rather hydrophobic protein [15] and forms interactions between subunits, partially via its well-conserved IXI sequence [16]. The short C-terminal extension, the region most distal in mammalian sHsps, is highly flexible and solvent exposed [17,18].

Human movement is controlled by the neurotransmitter, dopamine (DA), upon its release from neurons in the substantia nigra within the brain to the basal ganglia [19]. Parkinson’s disease (PD), a protein misfolding disease, is associated with reduction in cellular levels of DA due to the selective loss of dopaminergic neurons along with the abnormal oxidation of DA [20]. PD is also a disease of ageing, which concomitantly confers a decline in proteostasis [2]. DA is highly susceptible to oxidation at physiological pH [21,22]. In the cell, many mechanisms protect DA from oxidation such as sequestration within acidic vesicles and the presence of antioxidants such as glutathione (GSH) [21]. In PD, the substantia nigra completely lacks reduced GSH [23]. The plethora of species formed upon the oxidation of DA, e.g., aminochromes, can form adducts with nucleophilic side chains of amino acids such as cysteine, lysine and histidine [24,25,26] or interact non-covalently with amino acid sidechains via hydrogen bonding, π–π electron interactions, etc. Often, these interactions unfavorably affect the protein’s function. For example, oxidized DA intermediates form complexes with actin, α- and β-tubulin, condensing the cell’s cytoskeleton network [27]. Parkin, whose mutants are responsible for early onset PD [28], has its ubiquitin E3 ligase activity inactivated and its solubility diminished when covalently modified by oxidized DA (DA_ox_). Mutant forms of glucocerebrosidase are the most common risk factors for developing PD [29,30] and have decreased lysosomal hydrolase activity when incubated with DA [20]. α-Synuclein (αSyn) is the predominant protein found in the Lewy body brain deposits that are a characteristic feature of PD. αSyn is deposited in Lewy bodies in an amyloid fibrillar form. DA modifies αSyn by interacting with the C-terminal ^125^YEMPS^129^ region [31,32] and/or by forming covalent adducts [31,33,34]. Moreover, DA_ox_ stabilizes αSyn into its protofibril, oligomeric form, which is considered the most cytotoxic species during its aggregation [35,36]. In PD, αBc is upregulated in the substantia nigra [37] and αBc and Hsp27 are present in Lewy body deposits [38,39]. In vitro, αBc and Hsp27 inhibit amyloid fibril formation of αSyn [40,41]. Moreover, αBc and Hsp27 are components of neuromelanin granules in the substantia nigra, which progressively form with age and are primary storage sites for polymerized, oxidized DA [3]. Taken together, since αBc and Hsp27 are present in the dopaminergic neurons that are primarily affected in PD pathophysiology, they are potential targets for modification by DA_ox_.

Herein, we investigated, structurally and functionally, the interaction of DA_ox_ with αBc and Hsp27. Upon modification and cross-linking with DA_ox_ at an equivalent molar level, the sHsps were capable, to a degree comparable to that of the unmodified protein, of preventing amorphous and amyloid fibrillar protein aggregation, thereby corroborating the chaperones’ robust nature and their ability, in a cellular context, to mitigate the deleterious effects of protein unfolding and aggregation [5,6,7,42].

## 2. Results

### 2.1. Oxidized Dopamine-Modified sHsps Prevent Amorphous and Amyloid Fibrillar Protein Aggregation

Conventional methods to monitor the ability of DA_ox_-modified sHsps (sHsps:DA_ox_) to inhibit amyloid fibril and amorphous target protein aggregation could not be employed. For example, DA_ox_ quenches fluorescence of thioflavin T, the dye that binds to the β-sheet region of amyloid fibrils and is therefore used routinely to monitor amyloid fibril formation [43]. Furthermore, sHsps:DA_ox_ are large oligomeric species that scatter light, which skews turbidity measurements that are used to monitor amorphous protein aggregation.

Hence, a SDS-PAGE-based method was devised to monitor the soluble component of the aggregating protein over time, independent of sHsps:DA_ox_. The target protein was co-incubated with the sHsps:DA_ox_ under conditions that promoted unfolding and aggregation of the former. At each time point, the co-incubated sample was centrifuged to separate soluble protein from aggregated protein. Then, an aliquot of the soluble component (supernatant) was removed and flash frozen. Over time, a decrease in the concentration of soluble protein occurred due to the target protein aggregating. After the final time point, the frozen soluble components were thawed and run on SDS-PAGE. Due to lower mass of the aggregating target protein compared to the sHsps:DA_ox_, the band intensity of the target protein could be quantified and plotted against time. As expected, a decrease in the band intensity of the aggregating protein occurred over time, which enabled target protein aggregation to be monitored, without the interference of sHsps:DA_ox_. The normalized band intensities of the target proteins at the end of the experiment provided a comparison of the relative chaperone effectiveness of the native and DA_ox_-modified sHsps.

**Figure 1:**
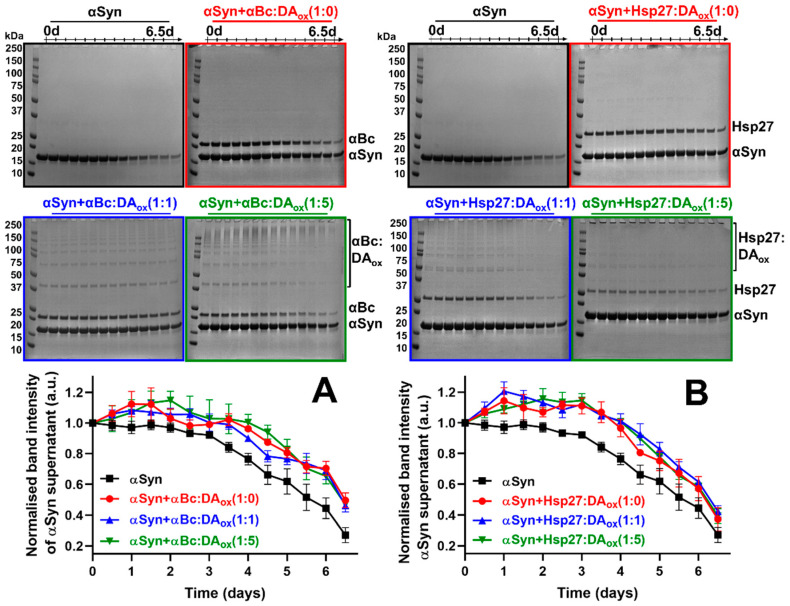

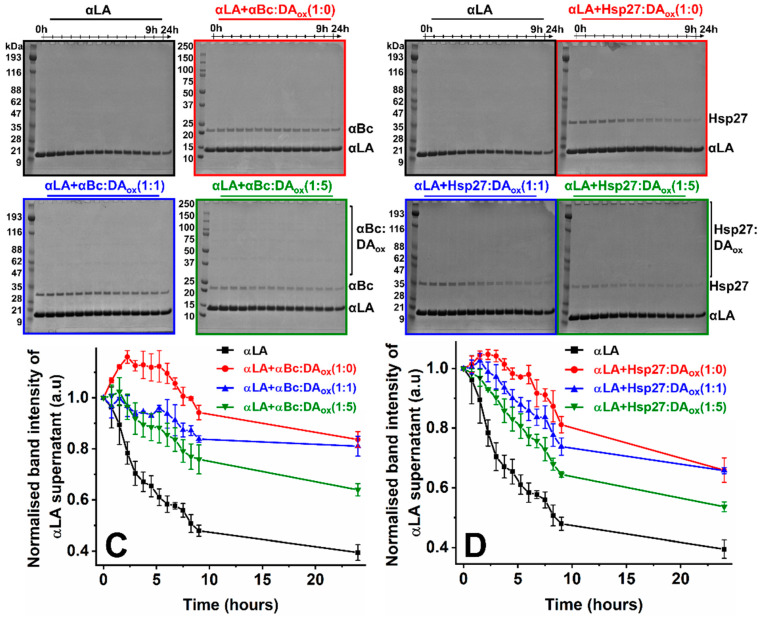
The effect of modification of αBc and Hsp27 by DA_ox_ on their chaperone activity against fibrillar and amorphously aggregating target proteins. 100 μM of A53T αSyn with or without DA_ox_-modified 10 μM αBc (**A**) or Hsp27 (**B**) was allowed to aggregate in 50 mM phosphate, 150 mM NaCl, pH 7.4 at 37 °C with shaking at 1500 rpm, in a total volume of 300 μL. Every ~12 h, the sHsp and αSyn co-incubation was centrifuged at 19,391 rcf for 15 min at room temperature. A volume of 15 μL of the supernatant was flash frozen and run on SDS-PAGE in a chronological order of time under standard conditions. The band at ~14 kDa in (**A**,**B**) reflects soluble monomeric αSyn and are data from one of the replicates. The band intensity was quantified using ImageJ software. The αSyn band intensity at each time point was normalized relative to the band intensity at 0 h. The data represent the average of three replicates. Error bars represent the standard error of the mean. 100 μM Apo αLA with or without DA_ox_-modified 4 μM αBc (**C**) or Hsp27 (**D**) was allowed to aggregate in 50 mM phosphate, 100 mM NaCl, 2.5 mM EDTA, pH 7.4 at 37 °C with shaking at 200 rpm. Every 45 min, the sHsp and αLA co-incubation was centrifuged at 19,391 rcf for 15 min at room temperature. A volume of 20 μL of the supernatant was flash frozen, and run on SDS-PAGE in a chronological order of time under standard conditions. The bands displayed in (**C**,**D**) reflect soluble monomeric αLA at ~14 kDa. The band intensity was then quantified using ImageJ software. The αLA band intensity at each time point was normalized relative to the band intensity at 0 h. The data represent the average of three replicates. Error bars represent the standard error of the mean.

The alanine to threonine point mutation at position 53 (A53T) in αSyn has been identified in patients with familial PD [44]. Under physiological conditions, A53T αSyn aggregates to form amyloid fibrils at a faster rate than the wild-type protein [40,41]. The aggregation of A53T αSyn was monitored over 6.5 days by SDS-PAGE, in the absence and presence of the native and DA_ox_-modified sHsps at a 10:1 molar ratio of A53T αSyn to the native sHsp or the sHsp modified with an equivalent or five-fold molar excess of DA_ox_ (e.g., αBc:DA_ox_ (1:5)). The complete gels for the time course of A53T αSyn aggregation in the presence of native and DA_ox_-modified sHsps are presented Figure 1A,B. As described above, in all cases, the band associated with soluble A53T αSyn (at ~14 kDa in mass) decreased with time. The other bands arise from αBc at ~20 kDa and Hsp27 at ~27 kDa along with their oligomeric forms (dimer, trimer, tetramer, etc.) due to cross-linking associated with reaction with DA_ox_. Large aggregates were present at the top of the gel which most likely arise from fibrillar A53T αSyn species and aggregated sHsps (more below). None of the gels exhibited intermediate species associated with A53T αSyn aggregation. Consistent with this, during chaperone action, our studies have shown that sHsps interact with the monomeric form of the target protein to prevent its association to form high molecular weight (HMW) species (e.g., [41,45]). When complete inhibition of the aggregation of an amyloid fibril-forming target protein (e.g., αSyn) occurs during chaperone interaction with sHsps, there are no HMW species formed, including a complex between the two [46].

Quantification of the reduction in the gel band from monomeric αSyn in the presence and absence of native and DA_ox_-modified sHsps is also shown in Figure 1A,B. Upon incubation of αBc:DA_ox_ with A53T αSyn, there was little difference (within the error of the experiment) between the chaperone ability of αBc modified with an equivalent and five-fold molar excess of DA_ox_ (αBc:DA_ox_ 1:1 and 1:5) compared to native αBc (αBc:DA_ox_ 1:0) (Figure 1A, Table 1). Similarly, the chaperone ability of unmodified Hsp27, or modified with DA_ox_ at the two molar ratios, to inhibit A53T αSyn aggregation was comparable within the error of the experiment (Figure 1B, Table 1). The chaperone ability of both sHsps modified with an equivalent and a five-fold molar excess of DA_ox_ (Supplementary Figure S1) and αBc modified with much higher molar equivalents of DA_ox_ (1:10, 1:20 and 1:30) (Supplementary Figure S2) was investigated over 24 h with another amyloid fibril-forming protein, reduced and carboxymethylated (RCM) α-lactalbumin (αLA). The highest ratio of αBc:DA_ox_ almost completely abrogated the chaperone ability of αBc (Supplementary Figure S2). Thus, large-scale modification of sHsps with DA_ox_ leads to impairment of sHsp chaperone ability against RCM αLA fibril formation whereas mild DA_ox_ modification causes little, if any, reduction in chaperone functionality against the two fibril-forming target proteins investigated.

Apo αLA undergoes well-characterized amorphous aggregation over 20-odd hours at physiological pH and temperature upon reduction of its four disulfide bonds [45]. When αBc which had been modified with DA_ox_ at an equimolar ratio was co-incubated with αLA, the aggregation of αLA was inhibited to an equivalent degree to that of native, unmodified αBc (i.e., αBc:DA (1:0)) (Figure 1C, Table 1). αBc modified with a five-fold higher concentration of DA_ox_ (αBc:DA_ox_ (1:5)) was still capable of inhibiting the aggregation of αLA but to a reduced degree compared to αBc:DA_ox_ (1:1), suggesting that greater modification of αBc by DA_ox_ decreases its chaperone ability. Upon Hsp27 modification with DA_ox_ at the same levels, a comparable trend was observed to αBc:DA_ox_ in its ability to inhibit the amorphous aggregation of αLA (Figure 1D, Table 1).

For both sHsps interacting with amyloid fibril-forming αSyn (Figure 1A,B) and RCM αLA (Supplementary Figures S1 and S2) and amorphously aggregating apo αLA (Figure 1C,D), a reduction in monomeric sHsps occurred with time, as monitored by densitometry (Supplementary Figure S3). For the DA_ox_-modified sHsps, some of this decrease in intensity arises from oligomerization of the sHsps due to cross-linking. Under the relatively rapid stirring conditions at pH 7 and 37 °C used for all these experiments (apart from those with RCM αLA), native αBc forms amyloid fibrils over 28 h (Kumar et al., unpublished results). Aggregation arises from shear effects that cause partial protein unfolding and amyloid fibril formation [47]. Consistent with this, mammalian sHsps, including αBc and Hsp27, have a high amyloid fibril-forming propensity, particularly within their ACD [48]. The day-long timeframe for sHsp fibril formation provides an explanation for the loss with time of native and DA_ox_-modified sHsps in the experiments summarized in Supplementary Figure S3. Our previous studies showed that in its amyloid fibrillar state, αBc retains, and in some cases increases, chaperone activity in preventing the amorphous and fibrillar aggregation of target proteins [49]. Thus, even though both sHsps form amyloid fibrils themselves during the time-course of the chaperone assays, their alteration in chaperone ability arises from DA_ox_ modification.

### 2.2. DA_ox_ Promotes Cross-Linking of sHsps

**Figure 2:**
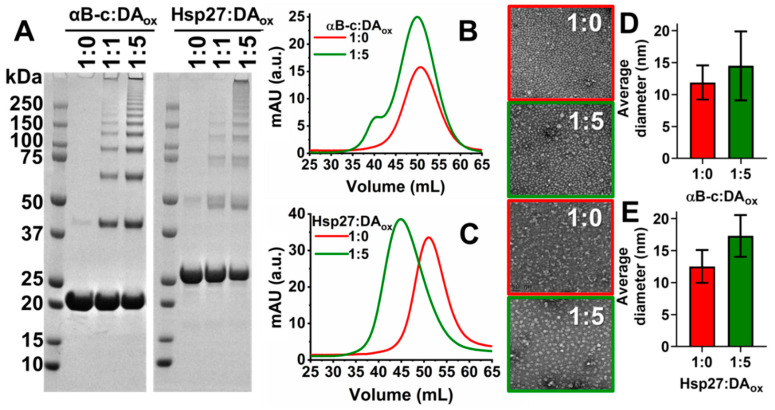
Characterization of DA_ox_-modified sHsps. (**A**) 15 μM of αBc or Hsp27 was incubated with 0, 15 or 75 μM of DA in 50 mM phosphate, 150 mM NaCl at pH 7.4 for 24 h at 37 °C. Subsequently, the excess DA_ox_ was removed by subjecting the sHsp and DA_ox_ to co-incubation in a 10 kDa cut-off centrifugal filter. This process was repeated five times with the same buffer. The protein was then run on SDS-PAGE. These gels are representative of at least three repeats. A volume of 1 mL of 40 μM of αBc (**B**) or Hsp27 (**C**) was reacted with 0 or 200 μM DA in 50 mM phosphate, 150 mM NaCl, pH 7.4 for 24 h at 37 °C. The samples were loaded on a HiPrep 16/10 Sephacryl S-300 HR gel filtration column, with a column volume of 120 mL and a molecular mass fractionation range of 10–1500 kDa. Negatively stained transmission electron microscopy (TEM) images of 30 μM αBc (**D**) and Hsp27 (**E**) when incubated with 0 or 150 μM DA_ox_. The diameter of 12 particles was measured per image using ImageJ software with nine images acquired per condition. The error bars represent the standard deviation.

The structural alterations to the sHsps as a result of DA_ox_ modification were investigated by a variety of biophysical methods. Via SDS-PAGE, DA_ox_ modification of αSyn leads to the formation of a well-defined, characteristic cross-link fingerprint of monomeric, dimeric, trimeric and larger oligomeric species [50]. The oligomers are not broken down into the monomer by SDS and boiling, consistent with covalent cross-links being responsible for oligomerization. Similarly, as determined by SDS-PAGE, both αBc:DA_ox_ and Hsp27:DA_ox_ formed higher molecular weight (HMW) species with a well-defined oligomer fingerprint of dimer, trimer, tetramer, etc. (Figure 2A, Table 2), a pattern that was not affected by the presence of an excess of the reducing agent, dithiothreitol (DTT) (Supplementary Figure S4). At a five-fold molar excess of DA_ox_ relative to the sHsps compared to an equivalent concentration of DA_ox_, SDS-PAGE showed that the monomeric band decreased in intensity concomitantly with an increase in intensity of the HMW bands. Thus, a higher concentration of DA_ox_ led to greater cross-linked oligomerization of the sHsps.

The oligomeric state of sHsps:DA_ox_ in solution, in the absence of the denaturant SDS, was investigated by size-exclusion chromatography. Using a buffer mimicking physiological conditions, sHsps:DA_ox_, particularly Hsp27, eluted from the size-exclusion column earlier than the native sHsps, consistent with them being larger in mass than the native sHsps (Figure 2B,C). Both sHsps:DA_ox_ exhibited an increase in intensity of their size-exclusion peak compared to their unmodified counterparts, which is attributed to the aromatic ring of DA_ox_ also absorbing at 280 nm [26], and being bound to and incorporated into the sHsps. Negative-stained transmission electron microscopy (TEM) revealed that there was an increase in the overall mean diameter of the αBc:DA_ox_ and Hsp27:DA_ox_ spherical oligomers in comparison to native αBc and Hsp27 (Figure 2D,E). For αBc, this increase in diameter was from 11.9 ± 2.7 nm to 14.5 ± 5.4 nm, and for Hsp27, the increase was from 12.5 ± 2.6 nm to 17.3 ± 3.3 nm upon modification with DA_ox_. The spherical morphology of the oligomeric sHsps was not altered upon modification with DA_ox_.

### 2.3. sHsps:DA_ox_ Retain Their β-Sheet Secondary Structure in the ACD

**Figure 3:**
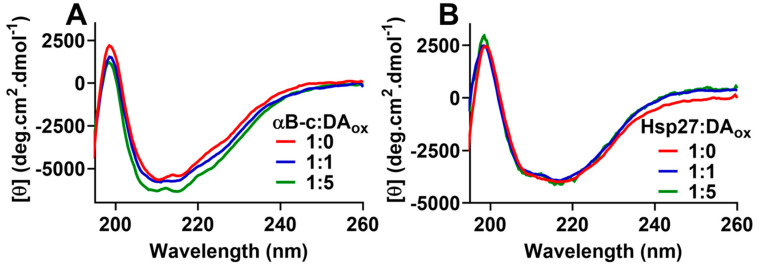
Reaction with DA_ox_ causes no significant change in the overall secondary structure of αBc and Hsp27. Far-UV CD spectra of (**A**) 8 μM αBc or (**B**) Hsp27 incubated with either 0, 8 or 40 μM of DA for 24 h. Prior to acquisition of the spectra, excess DA_ox_ was removed using a 10 kDa centrifugal filter. All spectra were acquired at 25 °C in 10 mM phosphate buffer, pH 7.4. The spectra displayed are representative of three replicates.

Characterization of sHsps:DA_ox_ using fluorescence spectroscopy, both intrinsic (i.e., fluorescence of the two tryptophan residues in the NTR of αBc) and extrinsic (i.e., 8-anilinonaphthalene-1-sulfonic acid (ANS) fluorescence to monitor exposed hydrophobicity), was not possible due to interference from the fluorescence of the incorporated DA_ox_ cross-links in the sHsps (Supplementary Figure S5). However, far-UV circular dichroism (CD) spectroscopy was used to monitor the effects of DA_ox_ on the overall secondary structure of the sHsps. The β-sheet-rich ACD of αBc interacts with amyloid-fibril forming proteins to prevent their aggregation [12]. Native sHsps have a CD spectrum indicative of the presence of significant anti-parallel β-sheet within the ACD, i.e., a positive peak at ~198 nm and a broad negative minimum at ~218 nm [51,52]. When both sHsps were modified with DA_ox_, there was no substantial change in the overall CD spectrum (Figure 3A,B), consistent with a retention of the β-sheet secondary structure in the ACD of αBc and Hsp27.

### 2.4. Lysine Residues Are Involved in the Formation of DA_ox_-Induced HMW Species of sHsps

As there was little change in β-sheet ACD secondary structure upon modification of the sHsps with DA_ox_, residues in the mostly unstructured NTR and CTR could be a target for DA_ox_. The highly solvent exposed, unstructured, flexible and short C-terminal extension in mammalian sHsps [17,18,53] is a prime candidate for modification by DA_ox_. As mentioned above, DA modifies a range of amino acids resulting in cross-linking of peptides and proteins [24]. In particular, the ε-amino group of the lysine side chain reacts with the catechol ring of a DA_ox_ intermediate [24,54], for example the DA *o*-quinone, to form a Schiff base linkage [55]. The C-terminal extension in both αBc and Hsp27 is well served with lysine residues, for example at their extreme C-terminus (Lys174 and Lys175 in αBc and Lys205 in Hsp27).

**Figure 4:**
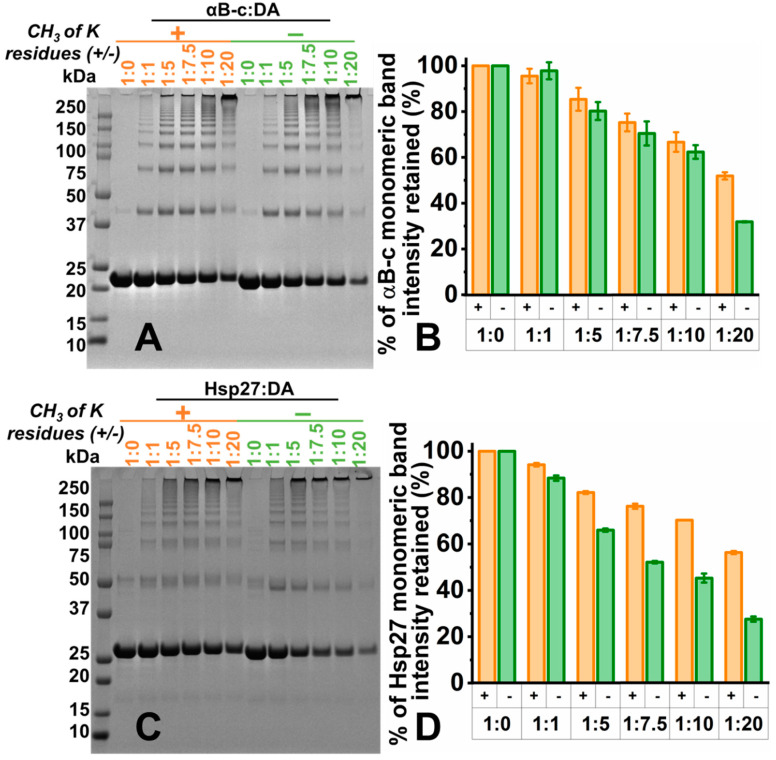
Lysine residues are involved in promoting HMW, cross-linked species of DA_ox_-modified αBc and Hsp27. After the selective dimethylation of lysine residues of αBc (**A**) and Hsp27 (**C**), they were reacted with a 0, 1, 5, 7.5, 10 or 20 molar excess of DA and incubated at 37 °C for 24 h in 50 mM phosphate buffer, pH 7.4. The samples were then run on SDS-PAGE. The average intensity of the monomer bands from three replicates for modified αBc (**B**) and Hsp27 (**D**) was quantified using ImageJ software. The monomer band for each concentration of DA_ox_ was normalized against the monomeric band of the native (non-modified) sHsps (1:0 sHsps:DA_ox_).

The lysine residues of αBc and Hsp27 were selectively dimethylated. The dimethylation of all lysine residues (and the amino terminus) of both sHsps was confirmed by mass spectrometry (Supplementary Table S1). The methylated sHsps were reacted with DA_ox_, as described above, to determine whether the sHsp lysine residues were involved in DA_ox_-mediated cross-linking (Figure 4). To determine the extent of cross-linking and hence HMW oligomerization induced by DA_ox_, the monomeric band intensity in SDS-PAGE was quantified. The intensity of the monomeric band retained upon modification was compared with that of the monomeric band intensity of the unmodified sHsps. For both methylated and non-methylated (native) sHsps, a gradual loss of the monomeric band intensity (and hence an increase in HMW species) was observed as the concentration of DA_ox_ increased. Methylated αBc retained comparable monomeric band intensities to its non-methylated counterpart up to a 10-fold molar excess of DA_ox_. At a 20-fold molar excess of DA_ox_, substantially more monomer was retained for methylated αBc compared to its non-methylated counterpart (Figure 4A,B). Methylated Hsp27 retained comparable monomeric band intensities up to a molar equivalent of DA_ox_. However, at a five-fold molar excess of DA_ox_, substantially more monomer was retained for methylated Hsp27 (Figure 4C,D). Similar to methylated αBc, significantly more of the monomeric band was retained when the lysine residues were dimethylated in comparison to non-methylated Hsp27 upon reaction with a 20-fold molar excess of DA_ox_. Overall, methylated Hsp27 was less susceptible to modification by DA_ox_ than methylated αBc. Thus, lysine methylation of the sHsps leads to less cross-linking due to reaction with DA_ox_, particularly at higher ratios of sHsp:DA_ox_, more so for Hsp27 than αBc. The implication is that lysine residues (via their ε-amino groups) are involved in reacting with DA_ox_ to form covalent cross-links resulting in HMW sHsp species.

### 2.5. Acidic pH and the Antioxidant, Glutathione, Rescue sHsps from DA_ox_-Induced Oligomerization

**Figure 5:**
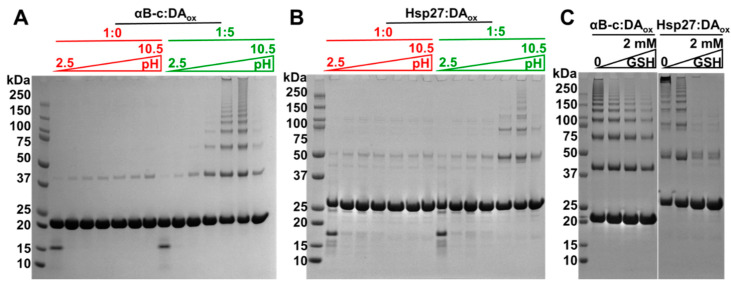
Acidic conditions and glutathione prevent formation of HMW, cross-linked sHsp species induced by DA_ox_. SDS-PAGE of 20 μM of αBc (**A**) and Hsp27 (**B**) modified with or without a five molar excess of DA_ox_ was incubated over a pH range from 2.5 to 10.5. (**C**) SDS-PAGE of 20 μM αBc and Hsp27 incubated with a five molar excess of DA_ox_ and 0, 0.1, 1 or 2 mM GSH in 50 mM phosphate, 150 mM NaCl for 24 h at 37 °C. The GSH was added immediately prior to commencing the sHsp-DA_ox_ interaction.

The protective mechanisms in the brain that prevent the oxidation of DA were mimicked in vitro to determine if they discouraged DA-induced cross-linking of sHsps. DA readily oxidizes at physiological pH. Hence, in vivo, DA is sequestered into acidic vesicles, thereby maintaining its protonated form [21,22]. The sHsps were reacted with DA in buffer at pH values of 2.5, 3.6, 4.8, 5.9, 7.1, 8.3 and 10.5, followed by examination via SDS-PAGE. At acidic pH (e.g., pH 2.5), no HMW sHsp species formed, i.e., only the monomeric form was observed (Figure 5A,B). With increasing pH, a greater number and intensity of HMW bands were observed for both sHsps. The HMW species were most prominent around physiological pH, i.e., at pH 7.1 and 8.3.

GSH is a reducing agent that is present at mM levels in the brain and other organs to protect from oxidative stressors such as DA_ox_. The concentration of antioxidants such as GSH declines with age, which contributes to the pathogenesis of diseases associated with oxidative stress including PD [23,56]. Accordingly, varying in vitro concentrations of GSH were co-incubated with the sHsps and DA at pH 7.4 prior to the formation of sHsps:DA_ox_ (Figure 5C). For both αBc:DA_ox_ and Hsp27:DA_ox_, substantially fewer HMW species were visible on SDS-PAGE in the presence of elevated levels of GSH. Thus, when 2 mM GSH was co-incubated with Hsp27 and DA_ox_, no species greater in mass than the dimer were observed. At the same concentration of GSH in the presence of αBc and DA_ox_, HMW species greater in mass than the dimer were visible via SDS-PAGE, but to a lesser extent in comparison to when the αBc:DA_ox_ co-incubation lacked GSH. Thus, for both sHsps, GSH at mM levels provided protection against DA_ox_ modification and cross-linking. GSH inhibits the oxidation of DA and hence its modification of the sHsps to form covalent cross-links. If formed, GSH does not dissociate the sHsp:DA_ox_ oligomers which are very stable as exemplified in Figure 2A where, even after treatment with a reducing agent (DTT), SDS and boiling, they do not dissociate. Similarly, Supplementary Figure S4 shows that the addition of DTT, at a 10-fold higher concentration in comparison to normal SDS-PAGE protocols, after the formation of the sHsp oligomers, did not lead to their dissociation.

The effect of GSH on αBc and Hsp27 should also be considered. In their native state, sHsps are highly dynamic, oligomeric species that are undergoing continuous subunit exchange, either via their monomeric or dimeric forms [5,6,7,8,9,10]. The presence of GSH in the surrounding medium is unlikely to affect the dissociation of sHsps. Most likely because of subunit exchange, DA_ox_ reacts with the dissociated sHsp species. GSH is unlikely to react with αBc as it does not contain any free sulfhydrl groups (it has no cysteine residues). Hsp27 has a single cysteine (Cys137) that could be glutathionylated. However, this is unlikely to have much effect on the protein as studies of modification at this site (e.g., mutation, S-thiolation and reductive methylation) did not reveal conformational change, let alone large alteration in the oligomeric state of Hsp27 [57].

### 2.6. DA_ox_-Modified sHsps Are Not Toxic to Cells

**Figure 6:**
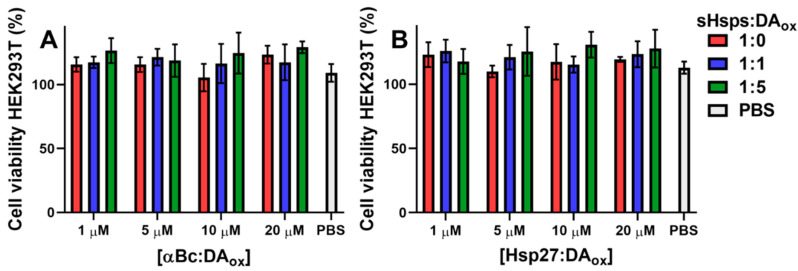
sHsps modified with DA_ox_ are not toxic to cells. 1, 5, 10 or 20 μM of αBc (**A**) or Hsp27 (**B**) was modified with a zero (red bars), one (blue bars) or five (green bars) molar equivalents of DA_ox_ and added as the final concentration in the growth media of HEK293T cells, and incubated for 24 h. The same volume of phosphate-buffered saline (PBS) was added as a control (grey bars). Cell viability was measured via dehydrogenase activity using a CCK8 kit. The viability percentages were normalized to cells without sHsps or PBS added. Values greater than 100% indicate increased cell viability relative to the controls due to the protective chaperone effect of sHsps on proteins. The bars represent an average of four replicates and error bars represent the standard deviation.

Finally, the effect of sHsps:DA_ox_ on cell viability was investigated. When HEK293T cells, which possess neuronal-like characteristics due to the presence of neurofilaments, vimentin and other proteins characteristic of a cell line with neuronal lineage [58], were treated with 1, 5, 10 and 20 μM of αBc:DA_ox_ (Figure 6A) or Hsp27:DA_ox_ (Figure 6B), there was no significant difference between cell viability in the presence of unmodified αBc or a phosphate-buffered saline (PBS) blank. The percentage values of cell viability were normalized to those of cells without sHsps or PBS added. The values in Figure 6 greater than 100% arise because the presence of sHsps increased cell viability relative to the controls, i.e., sHsps had a slight protective effect on the cells which is consistent with their ability as molecular chaperones to stabilize proteins. It is concluded that modification of sHsps with DA_ox_ did not induce cell toxicity over the experimental timeframe.

## 3. Discussion

PD is characterized by an increase in DA_ox_ [20] and protein aggregation, particularly of αSyn. Intracellularly, protein aggregation is mitigated by sHsps such as αBc and Hsp27 which form the first line of defense against proteostatic dysregulation. Of relevance to this study, sHsps are immunopositive in Lewy bodies and are overexpressed in disease states such as PD [37,38,39]. Despite the importance of sHsps in cellular proteostasis, how DA_ox_ affects their structure or function has not been investigated to date.

DA is inherently unstable in the presence of oxygen at physiological pH whereby it undergoes a cascade of reactions commencing with autoxidation leading to deprotonation of its hydroxyl groups. A further one electron oxidation leads to the formation of a DA *o-*semiquinone radical, which immediately forms a DA *o*-quinone and then undergoes cyclisation into an aminochrome. The aminochrome further reacts to form 5,6-dihydroxyindole, and then indole-5,6-quinone which self-polymerizes [21,22]. In the human brain, the polymerized DA_ox_ is the major component of neuromelanin, a dark pigment found within the cytoplasm of cells [21,59]. Neuromelanin also contains lipids and proteins related to protein degradation, autophagy, lysosomal and ubiquitin proteasome degradation systems including αBc and Hsp27, suggesting that neuromelanin is a storage site for these molecules which cannot be adequately degraded by the cell’s existing mechanisms [60,61]. Although the accumulation of neuromelanin progressively increases with age, in PD, there is a direct correlation between the amount of neuromelanin present in the cell and its death [62,63]. Hence, the oxidation of DA plays a key role in the pathogenesis of PD.

The present study demonstrated that the sHsps retain, to a significant degree, their chaperone activity after modification with DA_ox_ at equivalent or five-fold excess levels, against the amorphous aggregation of αLA and the amyloid fibrillar aggregation of A53T αSyn and RCM αLA. In general, chaperone ability was reduced at the higher level of DA_ox_ modification, more so at very high levels of DA_ox_ modification in preventing RCM αLA fibril formation. There is a plethora of evidence illustrating the reduction in protein function when modified with DA_ox_. For example, the E3 ligase activity of the protein parkin, a key enzyme in the proteosomal system, is inactivated upon modification with DA *o-*quinone [25]. In cells, upon the modification of actin and tubulin with aminochrome, the cytoskeleton of the cell was disrupted and microtubule polymerization was inhibited [27,64]. Most relevant to this study, αSyn forms unstructured adducts and oligomers upon the interaction with DA_ox_ [32,33,65,66,67]. Recently, there has been evidence suggesting that, in comparison to its unmodified counterpart, these αSyn oligomers are more effective at cross-seeding with Tau, a protein whose aggregation to form intracellular tangles is associated with Alzheimer’s disease [68], a process which may further exacerbate the disease’s pathogenesis. In a neuroblastoma cell line, PD-related proteins such as ubiquitin carboxy-terminal hydrolase L1 and DJ-1 were found to be conjugated with DA *o*-quinone [69]. In addition, a decrease in mitochondrial proteins such as MtCK and mitofilin occurred [69], which may cause a decrease in mitochondrial function to mitigate oxidative stress. Similarly, the structures formed during the oxidation of DA directly degenerate dopaminergic neurons [70] and induce neuroinflammation [71]. Hence, for αBc and Hsp27 to retain chaperone activity after cross-linking with DA_ox_ implies that they are highly robust chaperone proteins. Previous studies are consistent with this characteristic of sHsps. For example, when αBc was covalently bound to a solid-phase support [72] or formed amyloid fibrils [49], it still retained chaperone activity. However, the decrease in chaperone effectiveness of both sHsps at high molar excess of DA_ox_ implies that large-scale modification (cross-linking) of sHsps reduces their chaperone functionality.

When the sHsps:DA_ox_ were analyzed by SDS-PAGE after heating and the addition of reducing agent, they demonstrated a well-ordered pattern of monomer, dimer, trimer, tetramer, pentamer, etc. (Figure 2A, Table 2), as observed for other DA_ox_-modified proteins such as αSyn [50] and parkin [25]. Even after the addition of a 10-fold molar excess of DTT, the SDS-PAGE pattern was not altered (Supplementary Figure S4), i.e., the cross-linking was not affected by reducing agent. Moreover, as the concentration of DA_ox_ increased, the band intensity of the monomeric band for both sHsps decreased, and the higher molecular weight bands increased. The decrease in chaperone activity against αLA amorphous aggregation for the sHsps modified with a five-fold excess of DA_ox_ may be due to the decrease in concentration of the sHsp monomer, as the monomer is proposed to be the most chaperone-active species, compared to the sHsp oligomer [9]. The doubling of the Hsp27 HMW bands in SDS-PAGE (Figure 2A and Figure 4C) may be due the modification of Cys137 in the ACD of Hsp27. Cysteine residues are highly susceptible to modification by DA_ox_, in which a carbon atom of the DA *o-*quinone readily attacks the sulfhydrl group on the cysteine sidechain, forming a 5-S-cysteinyldopamine adduct [24,25,26]. In addition, αSyn, which does not contain a cysteine residue, was not as reactive towards DA o-quinone or other species in the DA oxidation cascade [26]. As αBc does not contain a cysteine residue, no doubling of its HMW bands was observed in SDS-PAGE (Figure 2A and Figure 4A). Cys137 is responsible for regulating Hsp27 dimerization, and responding to cellular oxidative stress [73,74]. DA adduct formation to this key cysteine may affect the dissociation of the Hsp27 oligomer, with a concomitant effect on its chaperone function and its regulation of cellular stress. There was no substantial alteration in the far-UV CD spectra of both sHsps upon modification with DA_ox_ (Figure 3), suggesting that the antiparallel β-sheet characteristics of the ACD remained intact, along with the structure of the mostly disordered NTR and CTR, which is consistent with the retention, predominantly, of chaperone activity upon mild DA_ox_ modification.

Both αBc:DA_ox_ and Hsp27:DA_ox_ eluted earlier from a size-exclusion column than their native counterparts, consistent with their larger size (Figure 2B,C). In agreement with this, TEM revealed that sHsps:DA_ox_ were larger in diameter than their native counterparts (Figure 2D,E). Moreover, there was no significant alteration in the spherical shape of the oligomeric sHsps upon modification with DA_ox_ compared to their native counterparts [75]. In contrast, the destabilized R120G mutant of αBc, which causes desmin-related myopathy, also has an increase in size as observed for αBc:DA_ox_ but has significantly reduced chaperone ability [76].

The CTR of mammalian sHsps has a flexible, unstructured C-terminal extension at its extremity which is highly solvent exposed [17,18,53,77], making this region potentially susceptible to reaction with DA_ox_. For both αBc and Hsp27, there are more lysine residues in the CTR in comparison to the NTR and the ACD; 23% and 8.7% of residues are lysine in the CTR of αBc and Hsp27 compared to 7.2% and 5.0%, respectively, in the ACD. Moreover, lysine is the final amino acid of both proteins. sHsps with dimethylated lysine residues were less susceptible to DA_ox_ modification (Figure 4). As mentioned above, lysine readily reacts via its ε-amino group with the DA *o-*quinone to form a Schiff base linkage [24,55]. Similarly, upon reaction of α-crystallin proteins with galactose, lysine residues in the C-terminal extension were covalently modified via a Schiff base linkage between the aldehyde group of galactose and the lysine ε-amino group [78], a modification which is common in the diabetic eye lens. In vivo, lysine residues of sHsps could react with and thereby quench damaging oxidants, and in doing so, provide another function for these proteins in maintaining proteostasis. DA_ox_ modification is not limited to lysine residues. Other amino acid residues are readily modified by DA *o-*quinone such as cysteine, methionine and histidine [24], with some being more reactive to one intermediate in the DA oxidation cascade than others [26]. Cys137 in the ACD of Hsp27 is a prime candidate for modification by DA_ox_. Thus, as discussed above, reaction of DA_ox_ with Cys137 in Hsp27 could have a similar redox-regulating capability [9]. Furthermore, adduct formation via DA_ox_ is not the only modification (e.g., hydrogen bonding) that could affect sHsps [24,33].

In vivo, cellular mechanisms which prevent DA oxidation potentially would discourage the formation of cross-linked, HMW species of sHsps:DA_ox_. Thus, DA secreted from the presynaptic terminals in neurons is immediately sequestered into strongly acidic, monoaminergic vesicles called VMAT2, ensuring that the hydroxyl groups of DA are fully protonated, and hence minimizing the possibility of oxidation [79]. Excess DA remaining in the cytosol, which is at neutral pH, is enzymatically degraded [21]. Consistent with this, in vitro, no cross-linked HMW sHsps formed at pH 2.5 in the presence of DA_ox_ (Figure 5A,B). Around pH 7.1 and 8.3, oligomer formation was most pronounced. GSH is present at mM levels in the brain to counteract the effects of oxidative stress. The presence of GSH at these levels decreased the in vitro formation of sHsps:DA_ox_ oligomers (Figure 5C), with Hsp27 exhibiting less susceptibility to modification by DA_ox_ than αBc. The effect of GSH in protecting against DA-induced cytotoxicity is apparent from a variety of studies. αSyn oligomers formed with GSH-conjugated aminochrome are non-toxic [80] in comparison to those formed in the presence of DA_ox_ [36]. In addition, GSH transferase, an enzyme which conjugates GSH to electrophilic compounds, protected a neuroblastoma cell line from aminochrome-induced neurotoxicity [81] and prevented degeneration in a mouse model of PD [82]. Conversely, DA *o*-quinone modifies and decreases the abundance of enzymes involved in GSH regulation [83], further exacerbating the susceptibility of the cell to oxidative stress. Consistent with these studies, sHsps:DA_ox_ are not toxic to neuronal-like cells (Figure 6).

In conclusion, the effect of DA_ox_ on proteins and biological processes has been experimentally challenging to study due to the plethora of species produced upon DA oxidation, their variation in reactivity with different amino acids, and the absence of neuromelanin in mice, despite a recent model which mimicked the levels of neuromelanin-like structures [84]. This study has demonstrated that at relatively low levels of DA_ox_ modification and cross-linking of αBc and Hsp27, chaperone activity was retained to a significant degree to prevent amyloid fibrillar and amorphous aggregation. Lysine residues are one of the sites of modification by DA_ox_. The sHsps, αBc and Hsp27, are key players in maintaining cellular proteostasis; they have resistance to oxidative stress, and their chaperone protective roles mitigate the deleterious effects of protein aggregation, including that of αSyn during PD pathogenesis.

## 4. Materials and Methods

### 4.1. Protein Expression and Purification

αBc, Hsp27 and A53T αSyn were expressed and purified as previously described [9,40,41].

### 4.2. Preparation of sHsps:DA_ox_

αBc and Hsp27 were incubated in 50 mM phosphate, 150 mM NaCl, pH 7.4 at 1:0, 1:0.5, 1:1, 1:2, 1:5, 1:10 and/or 1:20 molar ratios of DA HCl (Sigma) for 24 h at 37 °C under aerobic conditions to promote the oxidation of DA. The oxidation of DA was confirmed by the change in solution color from clear to brown-grey. The sHsp:DA mixture was then centrifuged at 19,391 rcf for 30 min at room temperature to remove any precipitates. The supernatant was transferred to a 0.5 mL 10 kDa mass cut off centrifugal filter to remove unreacted, excess DA. The filters were then spun at 14,000× *g* for 10 min. The eluate was discarded and the filters were filled with 50 mM phosphate, 150 mM NaCl, pH 7.4 and spun at 14,000× *g* for 10 min. This process was repeated five times before the filtrate was recovered. The filtrate was run on SDS-PAGE to confirm formation of the oligomeric, HMW sHsp species.

### 4.3. Chaperone Assays

Amorphous aggregation: 100 μM αLA (Sigma) with or without 4 μM αBc:DA_ox_ or Hsp27:DA_ox_ was allowed to aggregate in 50 mM phosphate, 100 mM NaCl, 2.5 mM EDTA, pH 7.4 at 37 °C with shaking at 200 rpm. Every 45 min, the sHsps and αLA co-incubation was centrifuged at 19,391 rcf for 15 min at room temperature. A volume of 20 μL of the supernatant was flash frozen, and run on SDS-PAGE in chronological order of time under standard conditions. The band intensity at ~14 kDa, reflective of soluble monomeric αLA, was quantified using ImageJ software. The αLA band intensity at each time point was normalized relative to the band intensity at 0 h. The data represent the average of three replicates. Error bars represent the standard error of the mean.

Amyloid fibrillar aggregation: 100 μM of monomeric A53T αSyn (previously 0.2 μm filtered and centrifuged to remove seeds) with or without 10 μM αBc:DA_ox_ or Hsp27:DA_ox_ was allowed to aggregate in 50 mM phosphate, 150 mM NaCl, pH 7.4 at 37 °C with shaking at 1500 rpm in a total volume of 300 μL. Every ~12 h, the sHsp and αSyn co-incubation was centrifuged at 19,391 rcf for 15 min at room temperature. A volume of 15 μL of the supernatant was flash frozen and run on SDS-PAGE in chronological order of time under standard conditions. The band intensity at ~14 kDa, reflective of soluble monomeric αSyn, was quantified using ImageJ software. The αSyn band intensity at each time point was normalized relative to the band intensity at 0 h. The data represent the average of three replicates. Error bars represent the standard error of the mean.

The amyloid fibrillar aggregation of RCM αLA in the absence and presence of αBc:DA_ox_ or Hsp27:DA_ox_ was undertaken as previously described [85].

### 4.4. SDS-PAGE

SDS-PAGE was performed using Bis-Tris 4–12% gradient gels (Invitrogen) in a MES running system. Each sample contained 6 x protein loading dye containing DTT and was boiled for 5 min at 95 °C. Precision Plus Protein^TM^ Dual Color Standards (Biorad) or the Triple-color Protein Ladder One (Product No. 09547-74, Nacalai Tesque) were used as the molecular weight standards. The gel was run for 60 min at 140 V and stained with Coomassie blue (0.1% *w*/*v* Brilliant Blue R, Sigma), 40% methanol, 10% acetic acid in water and de-stained in 40% methanol, 10% acetic acid in water.

### 4.5. Mass Determination of sHsp:DA Oligomers from SDS-PAGE

ImageJ software (NIH) was used to determine the relative motility value (R_f_) by dividing the *y*-axis value of the sHsp band by the *y*-axis value of the dye-front. A linear equation was fitted upon plotting the log_10_ mass (*x* axis) against R_f_ for the molecular weight standards from which the masses of the sHsps:DA_ox_ bands were estimated _._

### 4.6. Size-Exclusion Chromatography

1 mg/mL of sHsps:DA_ox_ was loaded on a HiPrep 16/10 Sephacryl S-300 HR gel filtration column (GE Healthcare) at a flow rate of 1 mL/min in 50 mM phosphate buffer and 150 mM NaCl, pH 7.4, and eluted over one column volume (120 mL). Absorbance was monitored at a wavelength of 280 nm.

### 4.7. Circular Dichroism Spectroscopy

The far-UV CD spectra of 10 μM of sHsps:DA_ox_ in 10 mM phosphate buffer, pH 7.4 at 37 °C were acquired from 190–260 nm, at a 0.5 nm interval (in a Spectrosil® Far-UV Quartz 21-Q-1 cuvette, Hellma). The total scan time was ~9 min. All spectra were acquired on an Applied Photophysics Chirascan spectrophotometer.

### 4.8. Cell Viability Assays

Cell Counting Kit-8 (Sigma) was performed according to the manufacturer’s guidelines. Briefly, cell viability was measured by the addition of WST-8, a compound which produces an orange formazan dye when reduced by cellular dehydrogenases. Production of the dye was assessed spectrophotometrically at a wavelength of 460 nm. HEK293T cells were grown in Dulbecco’s Modified Eagle’s Medium (DMEM)—high glucose, 10% *v/v* Fetal Bovine Serum (FBS), 1% *v/v* Penicillin and Streptomycin and 1% *v/v* Non-Essential Amino Acids. Three biological replicates were performed. The percentage of viability was calculated as:

$$\%\;viability=\;\frac{Measured\;absorbance\;of\;treated\;cells}{Median\;absorbance\;of\;untreated\;cells}\times \;100$$

### 4.9. pH-Based Incubations

DA_ox_-modified sHsps were prepared as described above. For the various pH values, the DA and sHsp reactions were performed in the following buffers: 50 mM glycine-HCl at pH 2.5 and 3.6, 50 mM sodium acetate at pH 4.8, 50 mM phosphate at pH 5.9 and 7.1, 50 mM Tris-HCl at pH 8.3 and 50 mM Glycine-NaOH at pH 10.5.

### 4.10. Glutathione Incubations

20 μM of αBc or Hsp27 with 20 μM of DA was incubated with 0, 100, 1000, 2000 μM GSH (Sigma) in 50 mM phosphate, 150 mM NaCl, pH 7.4 for 24 h at 37 °C. 20 μL aliquots of the sHsp, DA and GSH co-incubated mixtures were loaded on SDS-PAGE.

### 4.11. Selective Dimethylation of sHsp Lysine Residues via Reductive Alkylation

Selective dimethylation of sHsp lysine residues was performed according to the reductive alkylation kit protocol of Hampton Research. The protein concentration in the filtrate was quantified by measuring the absorbance at 280 nm. The extinction coefficients for αBc and Hsp27 were 19,000 [86] and 40,450 M^−1^cm^−1^, respectively, which were obtained from the bioinformatics tool, Expasy Protparam. Using the methylated sHsps, sHsps:DA_ox_ were prepared as described above.

### 4.12. Mass Spectrometry

Mass spectra of the methylated and native (non-methylated) sHsps were acquired on an Orbitrap Elite Hybrid Ion Trap-Orbitrap mass spectrometer coupled with an UltiMate 3000 UHPLC (Thermo Scientific, USA). Samples were prepared in 50 mM phosphate buffer with 0.1% *v/v* formic acid.

## Figures and Tables

**Table 1:** Quantification of the chaperone ability of sHsps, as determined via densitometry from the percentage of soluble A53T αSyn and apo αLA retained at the end of the experiment, in the absence and presence of native and DA_ox_-modified sHsps.

	Final % of Soluble A53T αSyn Retained	
	αBc ± SEM	Hsp27 ± SEM
A53T αSyn only	27.0 ± 4.9	
αSyn + sHsps:DA_ox_ (1:0)	49.6 ± 4.9	37.2 ± 7.6
αSyn + sHsps:DA_ox_ (1:1)	46.2 ± 4.1	42.3 ± 3.7
αSyn + sHsps:DA_ox_ (1:5)	46.9 ± 2.6	39.3 ± 4.9
	**Final % of Soluble αLA Retained**	
	αBc ± SEM	Hsp27 ± SEM
αLA only	39.5 ± 3.1	
αLA + sHsps:DA_ox_ (1:0)	83.6 ± 3.1	65.9 ± 4.1
αLA + sHsps:DA_ox_ (1:1)	81.0 ± 3.9	65.8 ± 1.0
αLA + sHsps:DA_ox_ (1:5)	64.0 ± 2.4	53.6 ± 1.7

**Table 2:** Estimated masses of SDS and DTT-treated DA_ox_-modified αBc and Hsp27, as determined by SDS-PAGE from three replicates.

	αBc		Hsp27	
	Estimated mass ± STDEV (kDa)	Actual mass (kDa)	Estimated mass ± STDEV (kDa)	Actual mass (kDa)
Monomer	21 ± 0.6	20.2	26 ± 0.1	22.8
Dimer	43 ± 0.6	40.4	50 ± 0.4	45.4
Trimer	64 ± 1.0	60.6	75 ± 0.6	68.1
Tetramer	82 ± 2.2	80.8	97 ± 2.6	90.8
Pentamer	94 ± 5.7	101.0	111 ± 0.6	113.5

N.B. The actual mass values do not include the additional mass associated with DA_ox_-derived cross-links between subunits of αBc and Hsp27.

## Data Availability

The data presented in this study are available in this article and in its supplementary material.

## Supplementary Materials

Supplementary Materials can be found at https://www.mdpi.com/article/10.3390/ijms22073700/s1. Figure S1. The effect of modification of αBc and Hsp27 by DAox on their chaperone activity against reduced and carboxymethylated α-lactalbumin (RCM αLA); Figure S2. The effect of modification of αBc by a 10, 20 and 30 molar excess of DAox on its chaperone activity against reduced and carboxymethylated α-lactalbumin (RCM αLA). Figure S3. Densitometry of sHsp monomeric band intensity upon incubation with amorphous or amyloid fibrillar aggregating target proteins; Figure S4. HMW DAox-modified Bc species are not affected by the presence of excess DTT. Figure S5. ANS fluorescence of DAox-modified sHsps. Table S1. Masses determined by electrospray mass spectrometry of methylated sHsps.

## Abbreviations

αBc	αB-crystallin
ACD	α-crystallin domain
ANS	8-anilino-1-napthalenesulfonic acid
αSyn	α-synuclein
CTR	C-terminal region
CV	Column volume
DA	Dopamine
DA_ox_	Oxidized dopamine
DTT	Dithiothreitol
GSH	Glutathione
HMW	High molecular weight
Hsp27	Heat-shock protein 27
NTR	N-terminal region
PD	Parkinson’s disease
rcf	Relative centrifugal force
R_f_	Relative motility
rpm	Revolutions per minute
TEM	Transmission electron microscopy
