# Supplementary material for: The Effect of Oxidized Dopamine on the Structure and Molecular Chaperone Function of the Small Heat-Shock Proteins, αB-Crystallin and Hsp27
Source: Int J Mol Sci. 2021 Apr 2;22(7):3700. doi: 10.3390/ijms22073700 (PMC8037807; doi:10.3390/ijms22073700)
Supplement: Supplementary file 1 [file ijms-22-03700-s001.pdf]

SUPPLEMENTARY INFORMATION

# The Effect of Oxidized Dopamine on the Structure and Molecular Chaperone Function of the Small Heat-Shock Proteins, αB-Crystallin and Hsp27

Junna Hayashi, Jennifer Ton, Sparsh Negi, Daniel E.K.M. Stephens, Dean L. Pountney, Thomas Preiss and John A. Carver

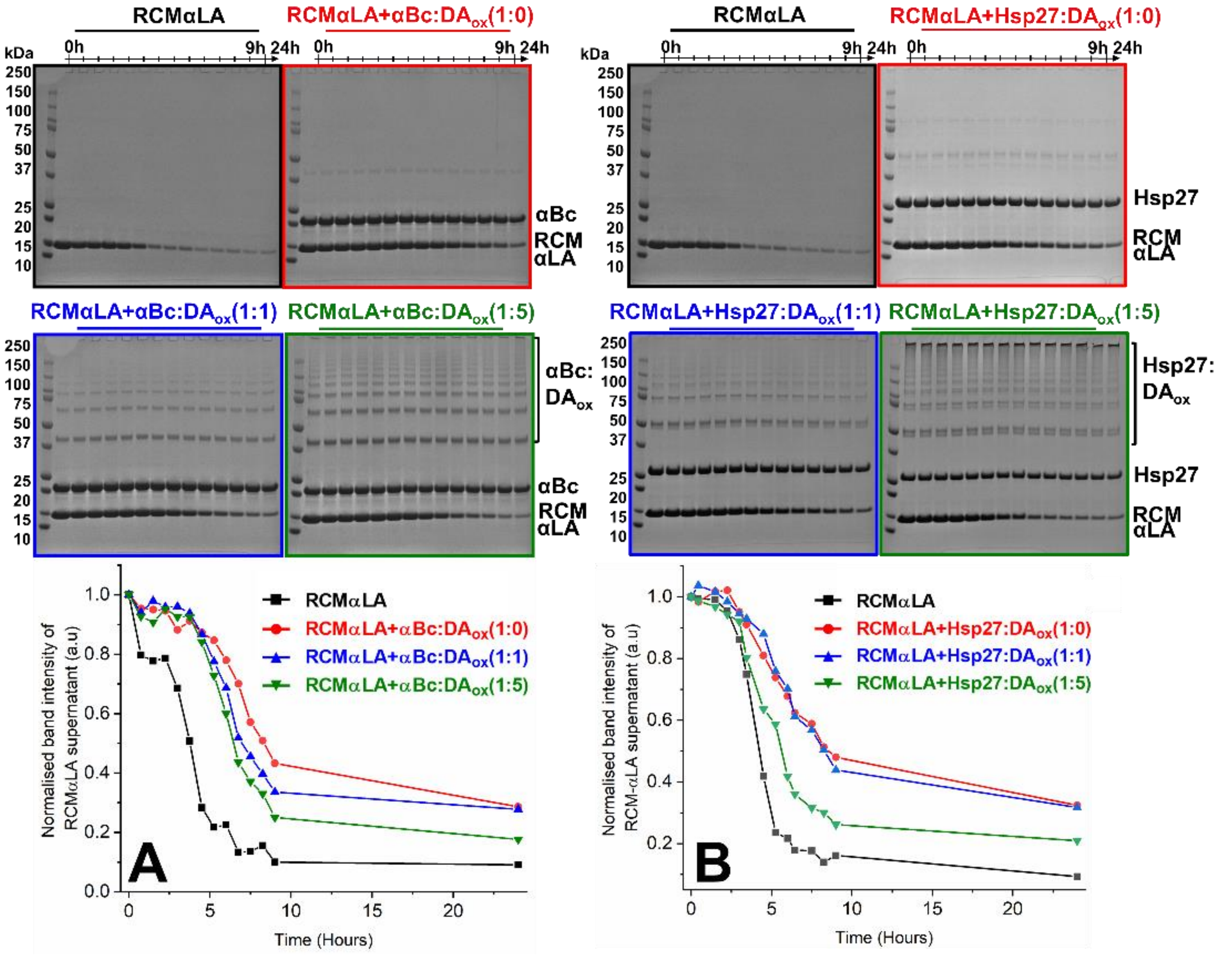


Supplementary Figure 1. The effect of modification of αBc and Hsp27 by $DA_{ox}$ on their chaperone activity against reduced and carboxymethylated α-lactalbumin (RCM αLA). αBc:$DA_{ox}$ (A) or Hsp27:$DA_{ox}$ (B) were prepared at a 1:0, 1:1 and 1:5 molar ratio of sHsps:$DA_{ox}$. Then, 30 µM RCM αLA was incubated with 10 µM of the respective sHsps:$DA_{ox}$, and allowed to aggregate, in 50 mM phosphate, 150 mM NaCl, 5 mM $MgCl_2$ at 37 ℃, pH 7.4 with no shaking. To separate the soluble and insoluble components, the incubation was centrifuged at 13,500 rpm for 15 min. 25 µL of the supernatant was taken and flash frozen. The samples were then loaded on SDS-PAGE. *SDS-PAGE gels.* The bands represent the soluble component of RCM αLA taken every 45 min until 9 h, and a final time point at 24 h. *Graphs:* The band intensity of RCM αLA was quantified using ImageJ software. The data were normalized against time 0 h.

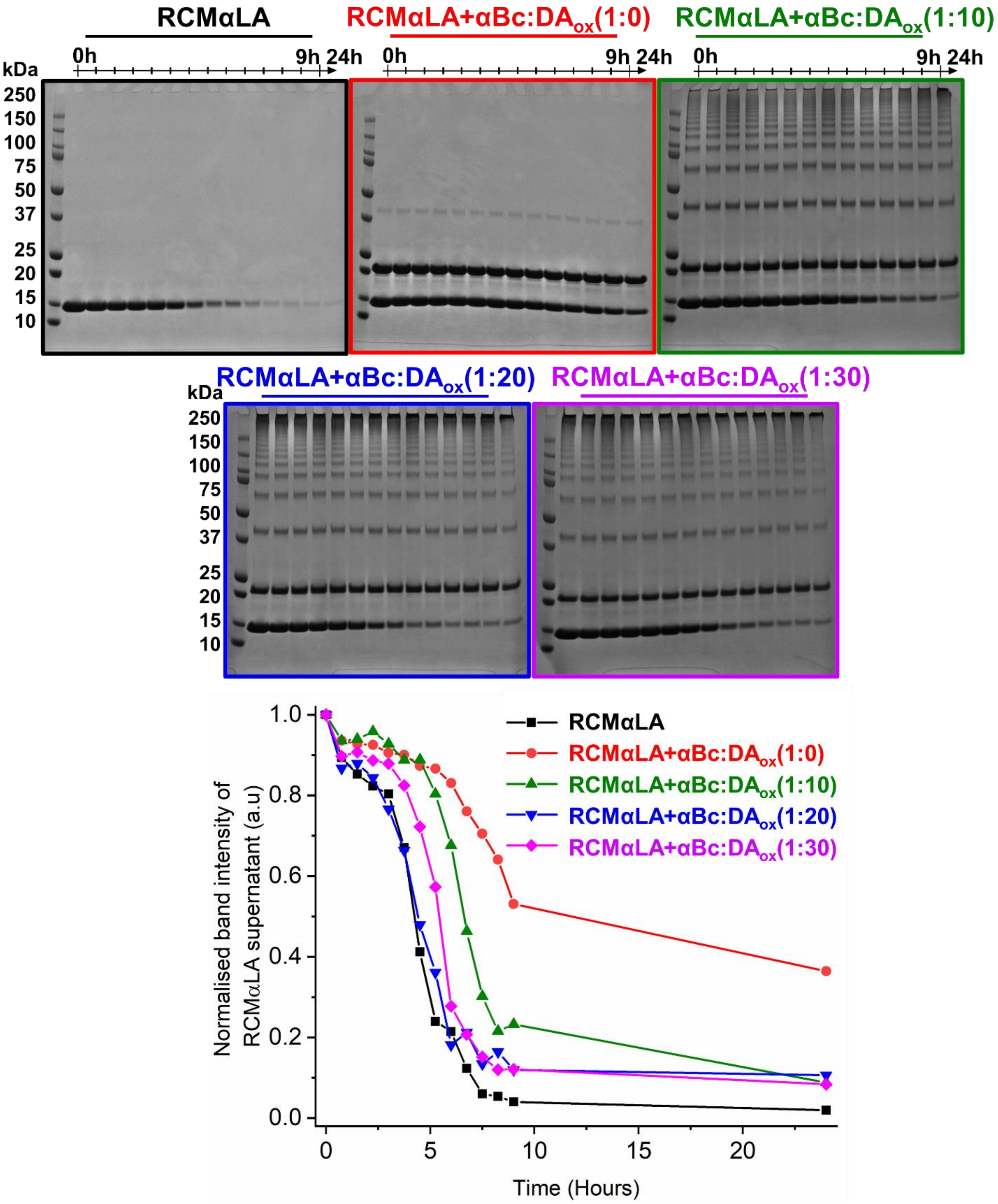


Supplementary Figure 2. The effect of modification of αBc by a 10, 20 and 30 molar excess of $DA_{ox}$ on its chaperone activity against reduced and carboxymethylated α-lactalbumin (RCM αLA). αBc:$DA_{ox}$ was prepared at a 1:0, 1:10, 1:20 and 1:30 molar ratio of sHsps:$DA_{ox}$. Then, 30 μM RCM αLA was incubated with 10 μM of the respective sHsps:$DA_{ox}$ and allowed to aggregate in 50 mM phosphate, 150 mM NaCl, 5 mM $MgCl_2$ at 37 ℃, pH 7.4 with no shaking. To separate the soluble and insoluble components, the incubation was centrifuged at 13,500 rpm for 15 min. 25 μL of the supernatant was taken and flash frozen. The samples were then loaded on SDS-PAGE. *SDS-PAGE gels:* The bands represent the soluble component of RCMαLA taken every 45 min until 9 h, and a final time point at 24 h. *Graph:* The band intensity of RCMαLA was quantified using ImageJ software. The data were normalized against time 0 h.

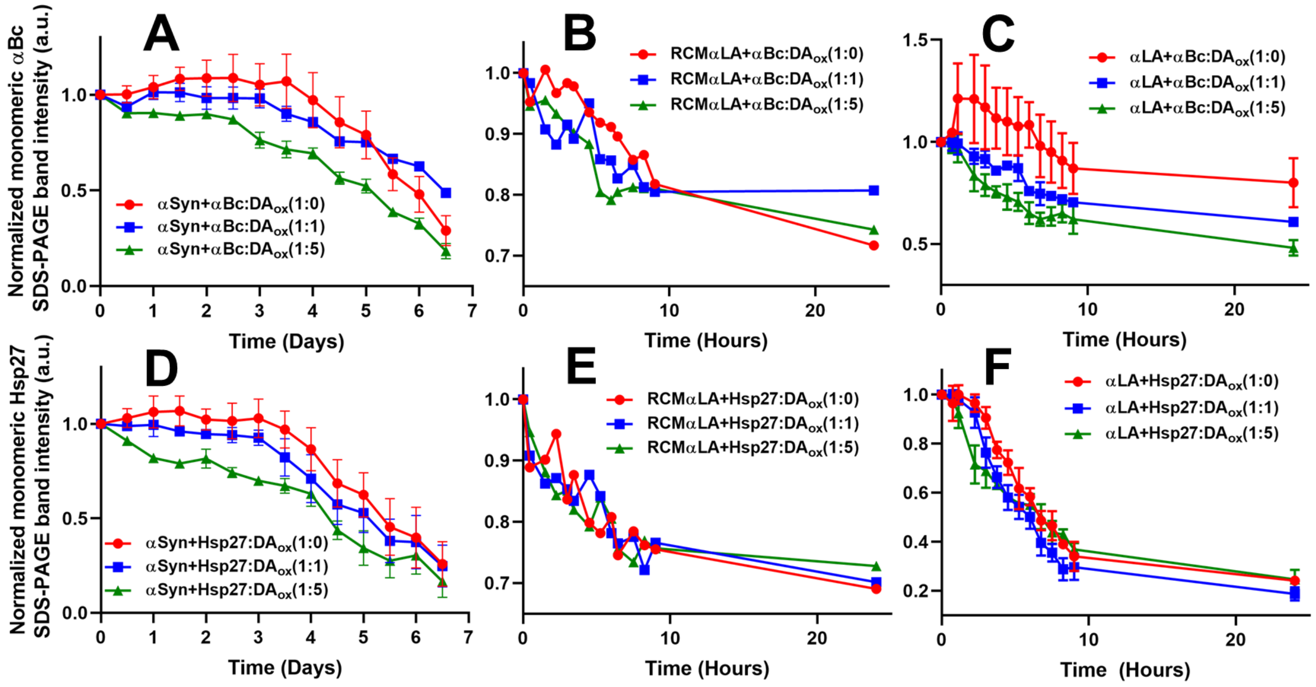


Supplementary Figure 3. Densitometry of sHsp monomeric band intensity upon incubation with amorphous or amyloid fibrillar aggregating target proteins. ImageJ software was used to quantify the band intensity of the sHsps:$DA_{ox}$ in the chaperone assays of Figures 1A, 1B, 1C and 1D, and Supplementary Figures 1A and 2B.

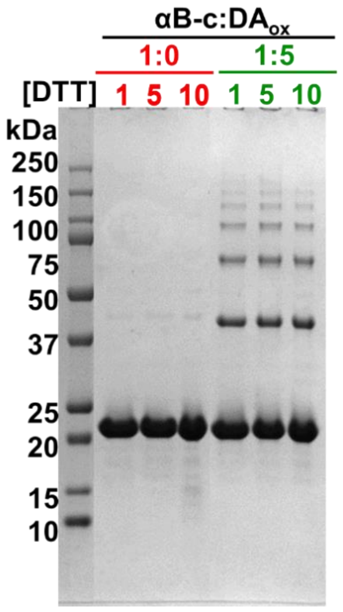


Supplementary Figure 4. HMW $DA_{ox}$-modified αBc species are not affected by the presence of excess DTT. 15 μM of αBc was modified with a five-fold molar excess of $DA_{ox}$. Prior to SDS-PAGE, the samples were additionally treated with a one, five or 10-fold molar excess of DTT.

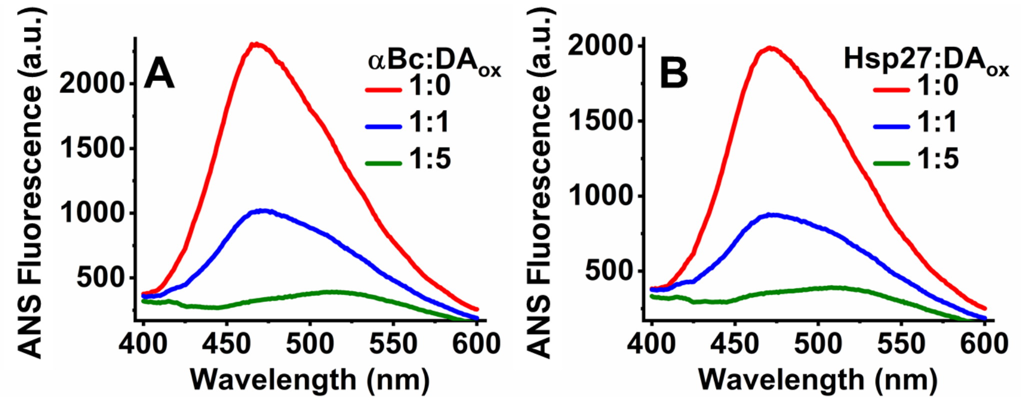


Supplementary Figure 5. ANS fluorescence of $DA_{ox}$-modified sHsps. ANS fluorescence spectra of 20 µM αBc:$DA_{ox}$ (A) or Hsp27:$DA_{ox}$ (B) after modification with zero, equivalent or five-fold excess of $DA_{ox}$ in the presence of 100 µM ANS in 10 mM phosphate buffer, pH 7.4 at room temperature. The displayed spectra are an average of three. The spectrum of 10 mM phosphate, pH 7.4, with 100 µM ANS was subtracted from each spectrum.

Supplementary Table 1. Masses determined by electrospray mass spectrometry of methylated sHsps

| | **Native (Da)** | **Methylated (Da)** | **Number of methyl groups added** |
|---|---|---|---|
| αBc | 20,158.4 | 20,466.7 | 22 |
| Hsp27 | 22,649.4 | 22,874.7 | 16 |

N.B. Dimethylation of the N-terminal amino group or of one lysine residue results in the addition of 28 Da in mass. Human αBc contains 10 lysine residues and one N-terminal amino group. Hsp27 contains seven lysine residues and one N-terminal amino group.
